# Comparative effectiveness of conservative and pharmacological interventions for chronic non-specific neck pain: Protocol of a systematic review and network meta-analysis

**DOI:** 10.1097/MD.0000000000016762

**Published:** 2019-08-16

**Authors:** Paolo Pillastrini, Greta Castellini, Alessandro Chiarotto, Francesco Fasciani, Francesco Marzioni, Carla Vanti, Lucia Bertozzi, Silvia Gianola

**Affiliations:** a Department of Biomedical and Neuromotor Sciences (DIBINEM) – University of Bologna, Italy; b IRCCS Istituto Ortopedico Galeazzi, Unit of Clinical Epidemiology, Milan; c Department of General Practice – Erasmus MC - University Medical Center Rotterdam; Department of Health Sciences – Amsterdam Movement Sciences Research Institute – VU University Amsterdam, the Netherlands.

**Keywords:** neck pain, network meta-analysis, rehabilitation, review, therapeutics

## Abstract

**Background:**

Neck Pain (NP) has been ranked as one of the top chronic pain conditions in terms of prevalence and years lived with disability in the latest Global Burden of Disease. NP has remarkable socio-economic consequences however, research efforts are limited. Discrepancies among guidelines recommendations on management of chronic neck pain exist. The purpose of this study protocol is to provide the methods for a review with network meta-analysis to identify the most effective interventions for chronic neck pain.

**Methods:**

The following databases will be searched from their inception to February 2019: Cochrane Controlled Trials Register (CENTRAL), PubMed, CINAHL, Scopus, ISI Web of Science and PEDro.

Randomized controlled trials (RCTs) on pharmacological and not pharmacological interventions will be included and their risk of bias will be evaluated using the Cochrane Risk of bias tool. Primary outcomes will be reduction in pain and disability. A network meta-analysis will be carried out and pairwise meta-analysis will be conducted using Stata 15 software. Grading of recommendations assessment, development, and evaluation (GRADE) will be applied to assess quality of the body of the evidence.

**Results:**

The results of this review will be submitted to a peer-review journal for publication.

**Conclusion:**

This network meta-analysis will provide a comprehensive review on the most effective treatments for the management of chronic neck pain providing key evidence-based information to patients, clinicians and other relevant stakeholders. Registration: PROSPERO (registration number CRD42019124501).

## Abstract 2

Supplemental Digital Content is available in the text

## 1 Introduction

Neck pain (NP) is listed among the worldwide leading causes for Years Lived with Disability according to the latest Global Burden of Disease 2017.^[1,2]^ NP has an annual prevalence ranging between 15% and 50%, being greater in females with the highest peaks in middle age.^[3]^ It has a multifactorial and complex etiology: it might be related to ergonomic or individual factors such as age, behavioral attitude or psychosocial distress such as anxiety or job satisfaction.^[4]^ The most common NP form is the non-specific which denotes pain not attributable to a specific cause. Usually, it is classified according to the duration of symptom into acute, when pain lasts less than 6 weeks, subacute when pain lasts up to three months or chronic when symptoms persist more than 3 months. Even though acute NP mostly resolves spontaneously (probably due to its natural course), nearly half of the patients will develop a chronic condition characterized by constant pain or frequent occurrences.^[5]^ The transition from acute to chronic is often related to biological, psychosocial, and occupational factors.^[6,7]^ The more persistent is the pain, the less favorable is its prognosis.^[8]^

The social and economic burden of NP is surely recognized^[9]^: quality of life, mood, ability to cope, social participation, employment rates and job income are reduced and influenced by NP both for who is affected by and their spouses. NP has also relevant consequences on productive capacity and sick leave, leading to high attributable costs.^[10]^ For instance, in the United States, costs in management for NP are estimated to be as high as $86 billion a year.^[11]^ Thus, it becomes fundamental to identify the most effective treatments for patient with chronic NP to reduce pain and disability.

Despite its known burden on families, communities, healthcare systems, and companies, NP has received very little attention in terms of research efforts, that is, 0.12 trials per million disability adjusted life years with a total of 30 million disability adjusted life years globally.^[12]^ Indeed, few large randomized clinical trials have focused merely on NP and evidence on how to manage this condition is often extrapolated from trials with mixed populations (e.g., spinal pain, chronic pain in general). Therefore, while several pharmacological and non-pharmacological intervention exist for the management of patients with chronic NP, there are discrepancies among guidelines recommendations: the most conflicting recommendations concern interventions such as electrotherapy, traction, laser therapy, acupuncture, heat/cold, and medications.^[13]^

The majority of the published clinical trials on the management of NP provide head-to-head comparisons of 2 interventions. Nevertheless, traditional pairwise meta-analyses are not able to integrate all the evidence from different studies with different comparisons. Recent methodological innovations from network meta-analysis (NMA) allow having a wider picture on effective interventions for a health condition by pooling results from individual studies and providing a multiple comparison evidence synthesis.

### 1.1 Aim

The goal of this study is to assess the effectiveness of currently available pharmacological and non-pharmacological interventions for patients with chronic non-specific NP. Moreover, the effectiveness of treatments for chronic NP will be ranked in respect to the likelihood to be the best among all the available treatments. To meet this goal, a comprehensive systematic review with a multiple comparison network meta-analysis will be performed. This review will shed light on the most effective treatments providing key evidence-based information to patients, clinicians and other relevant stakeholders (e.g., policy makers).

## 2 Methods

### 2.1 Protocol register

This systematic review protocol follows the Preferred Reporting Items for Systematic Review and Meta-Analyses Protocol (PRISMA-P) guidelines and its extension, PRISMA-NMA extension statement to compile network meta-analysis contents.^[14]^ We have completed the PRISMA-P checklist (Additional file 1). Our protocol has been registered in the PROSPERO database (PROSPERO 2019 CRD42019124501. Available from: http://www.crd.york.ac.uk/PROSPERO/display_record.php?ID=CRD42019124501).

### 2.2 Ethics

No ethical approval is needed since all the clinical studies included were approved by ethical committees and institutional review boards besides, patients signed a written informed consent.

### 2.3 Information sources and search strategies

We will search the following electronic databases from their inception to February 2019: Cochrane Controlled Trials Register (CENTRAL), PubMed (Medline), CINAHL, Scopus, ISI Web of Science and PEDro. The PubMed (Medline) search strategy, included in Appendix 1, will be adopted and adapted in the other databases. Hand manual search on non-indexed journal for further references will be performed. References list of all eligible studies and any systematic reviews and meta-analysis will also be checked during the search process. No restrictions regarding year of publication and language will be applied. In case of studies for which the English translation could not be made they will be classified as awaiting assessment.

## 3 Eligibility criteria

### 3.1 Study design

We will include only randomized controlled trials (RCT). Quasi-randomized trials and cross-over trials will be excluded.

### 3.2 Participants

We will include studies presented as full-text articles on adults (over 18 years old) with chronic non-specific NP. To be considered chronic, the pain should last for a minimum of 3 months duration at the time of intervention^[15]^ or, in the absence of this explicit description, when the authors themselves will define the pain as “chronic”. Consistent with the definition of non-specific NP, we will exclude studies including patients with

(i) specific diagnoses such as radicular pain, radiculopathy, myelopathy, fracture, infection, dystonia, tumor, inflammatory disease, and osteoporosis, and
(ii) subgroups of population (e.g., pregnant women). Studies focusing on whiplash-associated disorders or fibromyalgia will also be excluded as well as studies on mixed pain populations (e.g., spinal pain both from neck and back) where results for patients with NP are not presented separately.

### 3.3 Interventions

We will include single conservative intervention irrespective of modality (pharmacological and non-pharmacological), frequency or intensity, and treatment extent. We will include pharmacological interventions such as, for example, paracetamol, steroids, non-steroidal anti-inflammatory drugs (NSAIDs) or opioids; and non-pharmacological interventions like manual therapy, exercise therapy and so forth. We will exclude studies if the intervention will be surgical or an alternative form of medical treatments (e.g., homeopathy, herbal medicine).

**Figure 1:**
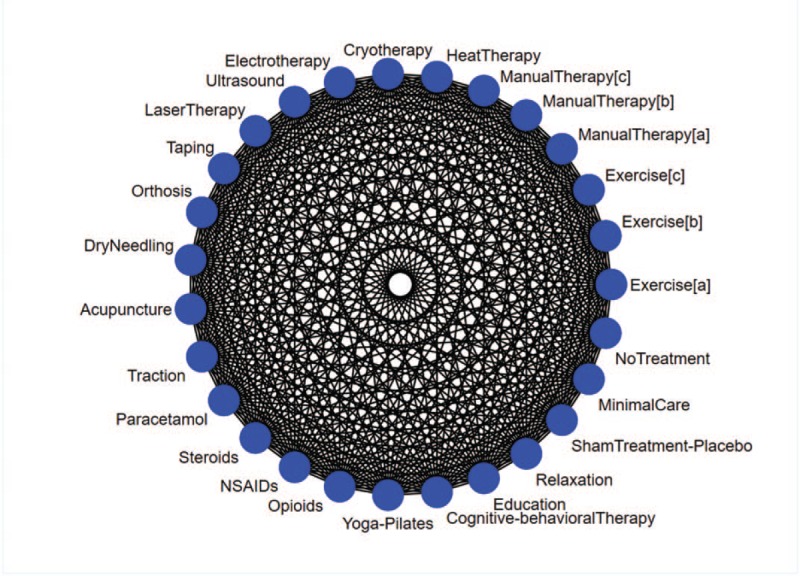
Network graph. Exercise [a]: Motor Control, Free and Supervised exercise; Exercise [b]: Strength and Endurance Training; Exercise [c]:Muscle Stretching. Manual Therapy [a]: Myofascial techniques, Trigger point treatment, Soft-tissue-techniques; Manual Therapy [b]: HVLA/Manipulation; Manual Therapy [c]: Passive mobilization, Mobilization-with-Movement.

Maximum 2 combined treatments will be considered as unique node according to a usual clinical practice (e.g., manual therapy plus exercise, manual therapy plus physical therapies, physical therapies plus exercise, exercise plus education).^[16]^ Since one of the major issues for non-pharmacological treatments is to gather a homogenous group for allowing comparisons, we will transparently justify the node-making process a posteriori once we obtain the eligible studies and assess the amount of information.^[17]^ However, a potential list of treatment involved in the nodes is reported in Figure 1.

### 3.4 Outcomes and study time-points

The primary outcomes of interest will be

(i) pain intensity measured, for example, with a Numeric Rating Scale (NRS) or a Visual Analogue Scale (VAS) and
(ii) physical functioning, that is, disability, measured, for example, with the Neck Disability Index (NDI) or the Neck Bournemouth Questionnaire (NBQ). These 2 outcomes have been consistently recommended as core outcome domains for clinical trials in patients with spinal pain.^[18,19]^ As secondary outcome we will report adverse events. We will gather information at the following time point: short-term (closest to 1 month assessment), intermediate (closest to 3–6 months) and long-term follow-up (closest to 12 months). For example, if a treatment lasted 3 weeks and the trial considers 1 week and 4 weeks of follow-up, we would select the 4 weeks as short term of point assessment.

### 3.5 Study selection

The list of abstract and title obtained by the searches will be screened according to inclusion and exclusion criteria by two independent authors. Then, the same 2 independent authors will assess full texts if potentially eligible. In case of disagreements, we will resolve through discussion or consulting a third author. Reasons for exclusion of full-texts will be monitored. We used Endnote (www.endnote.com) and Rayyan QCRI (REF https://rayyan.qcri.org/welcome) to manage the study selection phase.

### 3.6 Data extraction

We will use a pre-defined data extraction form within an Excel spreadsheet to gather the data form the included studies. Two authors will independently extract data about general characteristics and outcomes of interest from the included studies. Any disagreements will be solved via discussion with another member of the reviewing team.

We will extract the following general characteristics from each RCT: name of first author and country, year of publication, setting, number of centers, population characteristics (e.g., age, sex, pain duration), number of participants, dropouts, type of experimental and control intervention with details (e.g., length of treatment, frequency), primary and secondary outcomes’ measurements, signs of central sensitization. The outcome measures of interest will be gathered at post-treatment assessment. If any data from baseline and post treatment will not be available, we will contact the corresponding authors. In case of no response, missing values will be imputed from all outcome data as last option.^[20]^ Intention-to-treat analysis data will be used whenever available.

### 3.7 Risk of bias assessment

Risk of bias in RCTs will be assessed by Cochrane Collaboration's risk of bias tool *(modified version by the Cochrane Back & Neck*).^[21]^ Two authors will independently assess the domains as “low”, “high” or ‘unclear’ risk of bias if any information is reported. A summary ‘Risk of bias’ table will be incorporated into the interpretations of results. Any disagreements will be resolved by discussion and if consensus is not reached, another review author will be consulted and a decision made.

## 4 Data synthesis

### 4.1 Description of the available data

We will present the available direct evidence between different treatments performing a network diagram. We will evaluate the feasibility and the clinical relevance of the network graphically through the diagram and looking at

(i) the number of comparisons in the network with available direct data;
(ii) the presence of direct evidence based on single studies;
(iii) the presence of “closed loop”; and
(iv) whether any relevant treatment is not represented in the network.

### 4.2 Standard pairwise meta-analyses and network meta-analyses

We will perform a pairwise meta-analysis with at least 2 studies for each primary outcome using a random-effects model for each direct contrast.^[22]^ The I^2^ statistic will be used to explore between-study statistical heterogeneity within each head to head comparisons: I^2^ value of 25% to 49% will indicate low degree of heterogeneity, 50% to 75% moderate degree of heterogeneity and more than 75% high degree of heterogeneity.^[23]^ We will run these analyses using Review Manager 5.3 software.^[24]^

A network meta-analysis within a frequentist setting will be performed using all the available evidence. We will assume equal heterogeneity across all treatment comparisons, and accounting for correlations induced by multi-arm studies.^[25,26]^ We will use a multivariate normal model with random-effects ^[27,28]^ presenting all possible summary relative effect size for each outcome in a league table. We will also estimate the relative ranking of the effectiveness of pharmacological and non-pharmacological interventions through the distribution of the ranking probabilities and the surface under the cumulative ranking curves (SUCRAs). We will make use of Stata 15 software for the hereby described analyses, in particular we will use the ‘network’ package and results will be displayed with the network graph package.^[29,30,31]^

### 4.3 Assumption of transitivity

Transitivity is a fundamental assumption beyond the validity of indirect comparisons and, therefore, of the entire network meta-analysis. Thus, we will assume that any patient that meets the inclusion criteria is “jointly randomizable” to any of the eligible pharmacological and non-pharmacological treatment for NP in the network. We will assess the transitivity by looking at possible effect modifiers (clinical and methodological features) across the head-to-head comparisons.

### 4.4 Assessment of inconsistency

Consistency is the statistical manifestation of transitivity. We will evaluate inconsistency in the entire network using the design-by-treatment interaction model based on *χ* ^2^ test as described by Higgins et al.^[27]^ Then, we will look at local inconsistency in each closed loop using the loop-specific approach, which denotes the difference between direct and indirect estimates for a specific comparison (inconsistency factor). A node-splitting function will be also performed to separate trial-level evidence for a particular comparison (or node) to compare consistency from direct evidence (i.e., head-to-head trials from pairwise meta-analysis) versus indirect evidence (i.e., from network meta-analysis).

### 4.5 Subgroup and sensitivity analyses

If important heterogeneity and inconsistency are detected, we will explore the possible sources performing subgroup analysis.

### 4.6 GRADE quality assessment

We will use the Grades of Recommendation, Assessment, Development and Evaluation (GRADE) approach to evaluate the quality of the body of evidence contributed to the network.^[32,33]^ We will appraise the GRADE domains (study limitations, indirectness, inconsistency, imprecision and publication bias). The framework combines judgments about direct and indirect, providing an assessment of reliability of NMA treatment effects.

## 5 Discussion, ethics and dissemination

NP is one of the most burdensome musculoskeletal disorders in the adult population. Given its social and economic impact, it is urgent to find the most effective pharmacological or conservative treatments to reduce pain and improve physical functioning in people suffering from this condition. This systematic review is the first effort to compare direct and indirect effects of different conservative approaches in the management of patients with chronic NP. The results will have a direct positive impact on the development of future guidelines and on the clinical decision making process in treatment prescription. We will adopt strategies to disseminate our results to ensure the successful uptake of this research project, including peer-reviewed publications, conference presentations, dissemination to local, national, and international policy-makers.

## Author contributions

**Conceptualization:** Paolo Pillastrini, Alessandro Chiarotto, Carla Vanti, Silvia Gianola, Lucia Bertozzi.

**Data curation:** Francesco Fasciani, Francesco Marzioni, Carla Vanti, Lucia Bertozzi.

**Formal analysis:** Greta Castellini, Silvia Gianola.

**Investigation:** Francesco Fasciani, Francesco Marzioni, Carla Vanti.

**Methodology:** Greta Castellini, Alessandro Chiarotto, Silvia Gianola, Lucia Bertozzi.

**Project administration:** Paolo Pillastrini.

**Resources:** Greta Castellini, Silvia Gianola.

**Software:** Greta Castellini, Silvia Gianola.

**Supervision:** Paolo Pillastrini.

**Writing – original draft:** Greta Castellini, Carla Vanti, Silvia Gianola, Lucia Bertozzi.

**Writing – review & editing:** Greta Castellini, Alessandro Chiarotto, Carla Vanti, Silvia Gianola, Lucia Bertozzi.

Greta Castellini orcid: 0000-0002-3345-8187.

## Supplementary Material

Supplemental Digital Content
