# Supplementary material for: Comparative effectiveness of conservative and pharmacological interventions for chronic non-specific neck pain: Protocol of a systematic review and network meta-analysis
Source: Medicine (Baltimore). 2019 Aug 16;98(33):e16762. doi: 10.1097/MD.0000000000016762 (PMC6831373; doi:10.1097/MD.0000000000016762)
Supplement: Supplemental Digital Content [file medi-98-e16762-s001.docx]

**APPENDIX 1**

**Search strategy – Medline.**

((chronic [All Fields] AND ("neck pain"[MeSH Terms] OR ("neck"[All Fields] AND "pain"[All Fields]) OR "neck pain"[All Fields])) OR ((neckache OR neck ache OR cervicodynia OR cervicalgia OR cervicobrachial*) [tiab]) AND ((randomized controlled trials as topic[MeSH Terms]) OR (randomized controlled trial [All Fields] OR controlled clinical trial[All Fields] OR random* ))
